# Does third molar agenesis influence the second lower molar mineralization?

**DOI:** 10.1007/s00414-023-03128-5

**Published:** 2023-11-23

**Authors:** C. Ferreira, I. M. Caldas

**Affiliations:** 1 Faculdade de Medicina Dentária da Universidade do Porto, Rua Dr. Manuel Pereira da Silva, 4200-393 Porto, Portugal; 2 1H-TOXRUN–One Health Toxicology Research Unit, University Institute of Health Sciences, Gandra, Portugal; 3 Center for Functional Ecology-Science for People and the Planet (CFE), University of Coimbra, Coimbra, Portugal

**Keywords:** Agenesis, Dental age estimation, Forensic age estimation, Second molar, Tooth mineralization, Third molar agenesis

## Abstract

Different studies have established that the mineralization stages of the second mandibular molar can be used in forensic age estimation. Nowadays, the estimate’s accuracy is an ethical concern, producing as few false positives (individuals incorrectly classified as older than a determined threshold) and false negatives (individuals incorrectly classified as younger than a determined threshold) as possible. Some have hypothesized that changes in teeth number may influence tooth mineralization, altering the age estimate process. This paper analyzes whether third molar agenesis affects the second mandibular molar mineralization time frame. To do so, 355 orthopantomograms were evaluated for third molar agenesis, and the second mandibular molar mineralization stage was assessed using the Demirjian stages. Student’s *t*-test was used to compare the difference in the mean age at which the various stages of 37 mineralization were reached in the groups with and without third molar agenesis. The level of statistical significance was set at 5%. The results pointed to a delay in second mandibular molar mineralization in the case of agenesis, suggesting the need to consider this when estimating age using dental techniques.

## Introduction

In the last few years, ethical issues involving age estimation have been discussed [1–4]. Many authors address ethical issues related to the use of radiographic methods [5], but other questions, namely regarding the accuracy of the assessment, may arise. Focardi et al. [1] point out the necessity of the method of assessment being accurate, with as few false positives (individuals incorrectly classified as older than a determined threshold) and false negatives (individuals incorrectly classified as younger than a determined threshold) as possible. These concerns had also been raised by Garamendi et al. [6] who introduced the concept of ethical and technical errors regarding the overestimation and underestimation of age. In this manner, several attempts to enhance the accuracy of age estimation have been performed, and many pieces of research have been performed considering other factors that may affect dental age estimation techniques, namely socioeconomic status, population affinity, chromosomal anomalies, and diseases, among others [7–11]. The presence of agenesis, i.e., the congenital absence of teeth, has also been referred to as a variable to consider in age estimation [12, 13]. Dental agenesis or hypodontia is one of the most common anomalies in the development of human dentition [14] and is frequently associated with several other oral abnormalities [15–17]. The incidence of missing teeth may vary considerably depending on dentition and demographic or geographic profiles, and distinct patterns of agenesis have been detected in the permanent dentition (i.e., unilateral agenesis more common than bilateral; third molars (3M) and second pre-molars commonly missing in all quadrants and/or combinations of these two tooth types and the incisors) [14]. Sex also influences the prevalence rates of agenesis in both dentitions, which are significantly higher in females than males (3:2, respectively) [14].

As stated, dental agenesis also seems to be associated with delayed dental development. The permanent second mandibular molar (2M) erupts between 12 and 14 years of age. However, the root development is complete by 14–16 years of age. Multiple studies were carried out and confirmed the validity of the permanent mandibular second molar as an age marker in these age groups [18–21]. Yet, little is known about how tooth number variation can affect its mineralization process. This study aims to evaluate if the dental development of patients with agenesis is delayed, compared to a control group, particularly if there is an alteration in the pattern of mineralization of tooth 37 in individuals with agenesis of the 3M.

## Materials and methods

We have assessed 355 orthopantomograms (OPG) from patients from the Faculty of Dental Medicine of the University of Porto clinical services. The inclusion criteria were as follows: age between 9 and 15 years, Portuguese nationality, absence of a clinical history of mandibular third molar extraction, and having at least one intact mandibular second molar. Exclusion criteria were as follows: poor quality of OPG and evidence of pathology, local or systemic, and capable of influencing normal tooth development. OPGs were done for diagnostic purposes only, not specifically for this study. We collect data for two subsamples: S1, with no 3M agenesis, and S2, with individuals with at least one 3M agenesis. We have registered the age and sex of each participant. The total sample consisted of 187 males and 168 females, whose age distribution can be seen in Table 1. The age distribution by age group and sex is described in Table 2. The orthopantomograms were classified, assessing the presence of third molars and recording the mineralization phase using the stages proposed by Demirjian et al. [22].

**Table 1:** Age distribution of participants, in years

	Mean	Standard deviation	Maximum	Minimum
Males	12.42	1.34	14.98	9.90
Females	12.43	1.43	15.01	10.00

**Table 2:** Age distribution, in years, by age group, according to sex

Sex	Age group (years)	*n*	Mean	Standard deviation	Maximum	Minimum
Male	10	35	10.50	0.28	10.97	9.90
	11	39	11.56	0.25	11.90	11.03
	12	48	12.53	0.32	13.48	12.02
	13	36	13.42	0.29	13.98	13.01
	14	29	14.49	0.38	14.98	13.19
Female	10	35	10.48	0.31	11.09	10.00
	11	36	11.53	0.37	12.64	10.88
	12	26	12.44	0.29	12.98	12.00
	13	43	13.40	0.28	13.97	13.04
	14	28	14.52	0.31	15.01	13.97

S1 (*n* = 212) had mostly males (55.66%) aged between 10.15 and 14.98 years (mean = 12.47, standard deviation (sd) = 1.32); females’ mean age was 12.50 (sd = 1.33), ranging from 10.01 and 14.81 years.

As for S2, 40.3% had at least one tooth missing, affecting 69 (36.9%) males and 74 (44.0%) females. Table 3 depicts the number of third molar agenesis according to sex.

**Table 3:** Number of 3M according to sex (*n* = 236)

Number of 3M	Sex		Total (*n*)
	Male	Female	
0	15 (8.02)	11 (6.55)	26
1	13 (6.95)	12 (7.14)	25
2	20 (10.70)	21 (12.50)	41
3	21 (11.23)	30 (17.86)	51
4	118 (63.10)	94 (55.95)	212
Total (*n*)	187	168	355

Statistical analysis was performed using the Statistical Package for Social Sciences (SPSS) software, version 27.

Intra- and inter-observer errors were analyzed for the second molar mineralization stage, classifying a random sample of 20 OPG twice, by the same observer, with a 24-h gap, and by the two observers separately. Results were assessed using the Cohen’s kappa test. The inter-observer agreement showed substantial and significant agreement (kappa values of 0.882 (*p* < 0.001), and in the analysis of the intra-observer, the value obtained indicated a very high agreement, with *k* = 0.888 (*p* < 0.001).

Descriptive statistics were performed, with categorical variables described using frequencies and percentages and continuous variables using mean, standard deviation, and maximum and minimum values.

The chi-square test was used to verify possible associations between the agenesis of the third molars and the mineralization stage of the lower left second molar (37). The Spearman rank correlation was used to assess the correlation between the 3M agenesis number and 2M. Finally, Student’s *t*-test was used to compare the difference in the mean age at which the various stages of 37 mineralization were reached in the groups with and without third molar agenesis. The level of statistical significance was set at 5%.

The present study obtained a favorable recommendation from the Ethics Committee for Health of the FMDUP (recommendation n° 17/2022).

## Results

At least one 3M agenesis was found in 69 (36.9%) males and 74 females (44%). The frequency of agenesis by 3M is depicted in Table 4. It was found that, in males and females, the most frequent agenesis was the upper 3M (53.47% and 55.55%, respectively).

**Table 4:** Frequency of third molar agenesis, *n* (%)

Tooth	Agenesis	Males	Females
18	Yes	49 (26.2)	47 (28.0)
	No	138 (73.8)	121 (72.0)
28	Yes	37 (19.8)	43 (25.6)
	No	150 (80.2)	125 (74.4)
38	Yes	39 (20.9)	31 (18.5)
	No	148 (79.1)	137 (81.5)
48	Yes	35 (18.7)	31 (18.5)
	No	152 (81.3)	137 (81.5)

Table 5 shows the sample distribution concerning the 37 mineralization stages by sex and age. Stage G was the most frequent in males and females (50.8% and 47.0%, respectively). The earliest mineralization stage found was stage D in both sexes (0.5% in males and 1.2% in females). It was found that at 10 years old, stage F is more frequent; between 11 and 13 years, stage G is more recurrent; and at 14 years old, stage H is more common.

**Table 5:** Thirty-seven tooth mineralization stages by age group, *n* (males/females)

Stages	Age groups					Total (*n*)
	10	11	12	13	14	
D	1/2	0/0	0/0	0/0	0/0	1/2
E	14/11	6/2	3/1	0/0	0/0	23/14
F	15/14	15/10	12/5	1/2	2/1	45/32
G	5/8	18/24	32/17	27/21	13/9	95/79
H	0/0	0/0	1/3	8/20	14/18	23/41
Total	35/35	39/36	48/26	36/43	29”8	187/168

Third molar agenesis had a statistically significant association with second molar mineralization stages in both sexes (*p* = 0.049 and *p* = 0.028 for males and females, respectively). In both sexes, equal or more advanced maturation stages were seen in individuals without agenesis.

The maxillary 3M agenesis was associated with 37 mineralization stages in the age groups of 13 and 14 years old (*p* = 0.05 and *p* < 0.001, respectively) in males. As for females, there was a statistically significant association with 37 stages only in the age group of 11 years old. As for mandibular agenesis, there was a significant association with 37 stages in the 14-year-old males (*p* = 0.002) and with the 13-year-old age females (*p* = 0.015).

The number of 3M agenesis had a low but statistically significant negative correlation coefficient with 2M stage attainment in both sexes (Table 6).

**Table 6:** Correlation between the number of agenesis and 37 stage attainment

Number of 3M agenesis	Sex	
	Male	Female
Spearman’s rho coefficient	−0.299	−0.345
*p*	0.013	0.003

As for the difference between the mean age at the different 37 mineralization stages, only stage G attainment, both for boys and girls, depicted differences (*p* < 0.05) between both groups, with the children with agenesis displaying a higher mean age of stage attainment (Table 7). Stage D was not assessed since both sexes had no S1 (no agenesis) cases.

**Table 7:** Differences between groups (without -S1- or with -S2- 3M agenesis) regarding the mean age (in years) of 37 stage attainment in both sexes (*sd*, standard deviation)

Stage	Sex	Mean age S1 ± sd	Mean age S1 ± sd	Mean difference (S1-S2)	*p*
E	Male	10.79 ± 0.66	11.00 ± 0.86	−0.22	0.517
	Female	10.22 ± 0.019	10.69 ± 0.75	−0.47	0.321
F	Male	11.44 ± 0.79	11.86 ± 1.30	−0.42	0.185
	Female	11.31 ± 0.94	11.41 ± 1.2	−0.10	0.799
G	Male	12.57 ± 1.01	13.20 ± 0.98	−0.62	0.040
	Female	12.29 ± 1.12	12.83 ± 1.11	−0.55	0.037
H	Male	14.09 ± 0.80	13.25*	−0.08	0.313
	Female	13.74 ± 0.68	14.09 ± 0.82	−0.035	0.162

*Single case—no sd available

## Discussion

Dental maturity has been considered a suitable indicator of chronological age and regarded as superior to the stature and skeletal methods for evaluating an individual’s somatic maturity. So, it is considered a valuable tool to estimate biological age in forensic or archaeological studies. Yet, for accuracy improvement, other factors have been considered important when applying dental age estimation techniques, and agenesis is thought to be one of those factors.

Our study points to delayed dental maturation of the second mandibular left molar in individuals with at least one 3M agenesis, being this effect more pronounced as the number of 3M agenesis increases. This result agrees with the one obtained by Lebbe et al. [23] pointing out that the total number of agenesis needs to be considered when estimating age, as having more teeth agenesis is negatively correlated with dental age.

The effect of each tooth is, however, not clear. The maxillary 3M agenesis was associated with 37 mineralization stages in the 13- and 14-year-old males, and in females, only with the 11-year-old. As for mandibular agenesis, there was a significant association in 14-year-old males and 13-year-old females. Thus, maxillary agenesis may have an effect earlier than mandibular agenesis, and the effects of 3M agenesis are perceived in girls first.

This influence is difficult to explain. It may be that, at some ages, mineralization is more susceptible to congenitally absent teeth, making some stages more prone to this influence. The results comparing mean age in both groups (S1 and S2) support this hypothesis as stage G was the only one to depict statistically significant differences. In our case, the more susceptible ages, based on the greater number of associations, are 11 years old for females and 14 years old for males, which, in the Portuguese population, coincides with the pre-puberty ages [24, 25].

As for other studies, to date, to the best of our knowledge, this is the first study analyzing the third molar agenesis impact on second mandibular molar mineralization. Other investigators addressed delayed mineralization of permanent canines, premolars, and second molars in individuals with missing teeth, not including third molars [13, 26]. Daugaard et al. found that the second premolar unilateral absence is associated with a tooth mineralization delay on the same and the contralateral side [13]. Our research did not study the second lower right molar mineralization stages, so we cannot assess the described effect. The same authors also reported a relationship between missing second premolars and delayed second molars in girls but not boys. Our investigation does not confirm their findings, as the effect was seen in both sexes; Ruiz-Mealin et al. [26] did not also report sex differences in dental maturation delay in patients with agenesis. Yet, our results point to more robust differences between groups in females (*p* = 0.028 and *p* = 0.049, for females and males, respectively). This latter finding of differential effects in females and males may be of particular interest concerning sex differences in dental development, and the fact that, in our sample, the effects seem to be more pronounced in females suggests that this is most likely a substantial finding and not merely a consequence of multiple statistical tests.

Further studies also account for this effect of delayed teeth mineralization. Tunç et al. [27] reported a mean difference of the estimated dental age of 0.3 years between the agenesis and the control group. Similar results were obtained by other authors [28–30], who also reported the differences being more expressive in females.

The relevance of performing this study concerning specifically the second molar mineralization relates to the fact that, in some countries, 14 is the limit of criminal liability. For instance, in Italy, the subject may be liable from 14 to 18 years, depending on the degree of maturity reached [1]. Other reasons may relate to age estimation of victims of sex crimes. In Portugal, the 172nd article of the Portuguese Penal Code determines different sentences for abuse of minors if they are over or under 14 years of age [20, 21]. Regardless, the underestimation or overestimation of age in this age group caused by technical errors may conflict with ethical issues since the penalties applied will be inadequate. The error of the estimated age being different from the real age becomes even more relevant in child abuse or child pornography scenarios since the punishments are more severe in case the victim is younger than 14 years old [20].

Another point we would like to address is the methodology of 37 staging. Being a two-rooted tooth, and because the mineralization process does not happen simultaneously in both roots, we have staged 37 according to the root that displayed the more advanced mineralization stage (i.e., the one that would give the individual the higher age). We recognize that usually, it is the other way around. Yet, in this case, a higher age estimation, i.e., being older, leads to a better (more favorable) penal measure for the offender. In this way, we believe that by doing so, we favored technical and not ethical errors.

As for limitations, we cannot tell if our sample with no agenesis depicts a “normal development rate of the second molar,” but as no other factors were identified, we have assumed it does. Still, other factors can modify stage attainment [7, 9]. Nevertheless, when compared with a study in a similar population [20], our non-agenesis subsample depicted similar Demirjian’s stage attainment ages.

We also recognize that by using the Demirjian stages (or any other), we introduce large uncertainties since the assigned stage is attained, and suddenly, it goes from one stage to the next. Still being so widely used, it allows for comparison with other studies; additionally, stages (particularly Demirjian stages) are frequently used as markers or thresholds for given ages [31–33], and in this sense, this study can benefit those practices.

Finally, we recognize that the effect is discrete, and the differences in the development of tooth 37 are minor. If a *p*-value 0.01 instead of 0.05 was adopted, not so much would be significant.

## Conclusions

The results indicate a delay in the development and calcification of the second molar when there is agenesis of a third molar. This effect is slight and more pronounced in females, increasing with the number of 3M agenesis. This must be taken into consideration when dealing with the 14-year-old thresholds, as dental age estimates can, in these situations, lead to an underestimation of age and, in child abuse, to a more severe penal measure for the offender.
